# ^212^Pb in targeted radionuclide therapy: a review

**DOI:** 10.1186/s41181-025-00362-7

**Published:** 2025-07-01

**Authors:** Jarred Michael Scaffidi-Muta, Andrew David Abell

**Affiliations:** School of Physics, Chemistry and Earth Sciences, The University of Adelaide, Adelaide, SA 5005 Australia

**Keywords:** Lead-212, Bismuth-212, Targeted radionuclide therapy (TRT), Targeted alpha therapy (TAT), Cancer, Alpha radiation, Radiotherapy, Radiopharmaceuticals

## Abstract

**Background:**

The selective delivery of α-emitting radionuclides is emerging as a highly effective form of cancer therapy. With a short range and high cytotoxicity, α-particles can selectively kill cancerous cells whilst minimising harm to surrounding healthy tissue. As the parent of the α-emitter ^212^Bi, ^212^Pb has seen increasing therapeutic use on account of its favourable chemistry, half-life, and decay properties. This review comprehensively discusses the clinical development of ^212^Pb in recent years, particularly its production, chelation chemistry, and therapeutic adoption.

**Main body:**

Improvements in generator technology and supply have overcome the historically limited availability of ^212^Pb, enabling a surge of research in the field. Numerous bifunctional chelators have since been developed, which enable facile conjugation of ^212^Pb to a plethora of tumour targeting carriers. Advancements in nuclear imaging techniques, and the use ^203^Pb as an imaging surrogate, have enabled accurate biodistribution and dosimetry information to inform preclinical studies. These factors have attracted considerable commercial interest in ^212^Pb, culminating in the rapid translation of this radionuclide into the clinic, where it is being investigated in the treatment of a range of malignancies.

**Conclusion:**

Radiotherapy with ^212^Pb has shown enormous promise in preclinical and clinical studies. While challenges still remain before ^212^Pb can be more widely adopted, remarkable progress has been made in addressing these. At present, the therapeutic potential of ^212^Pb is only beginning to be realised.

## Background

**Fig. 1:**
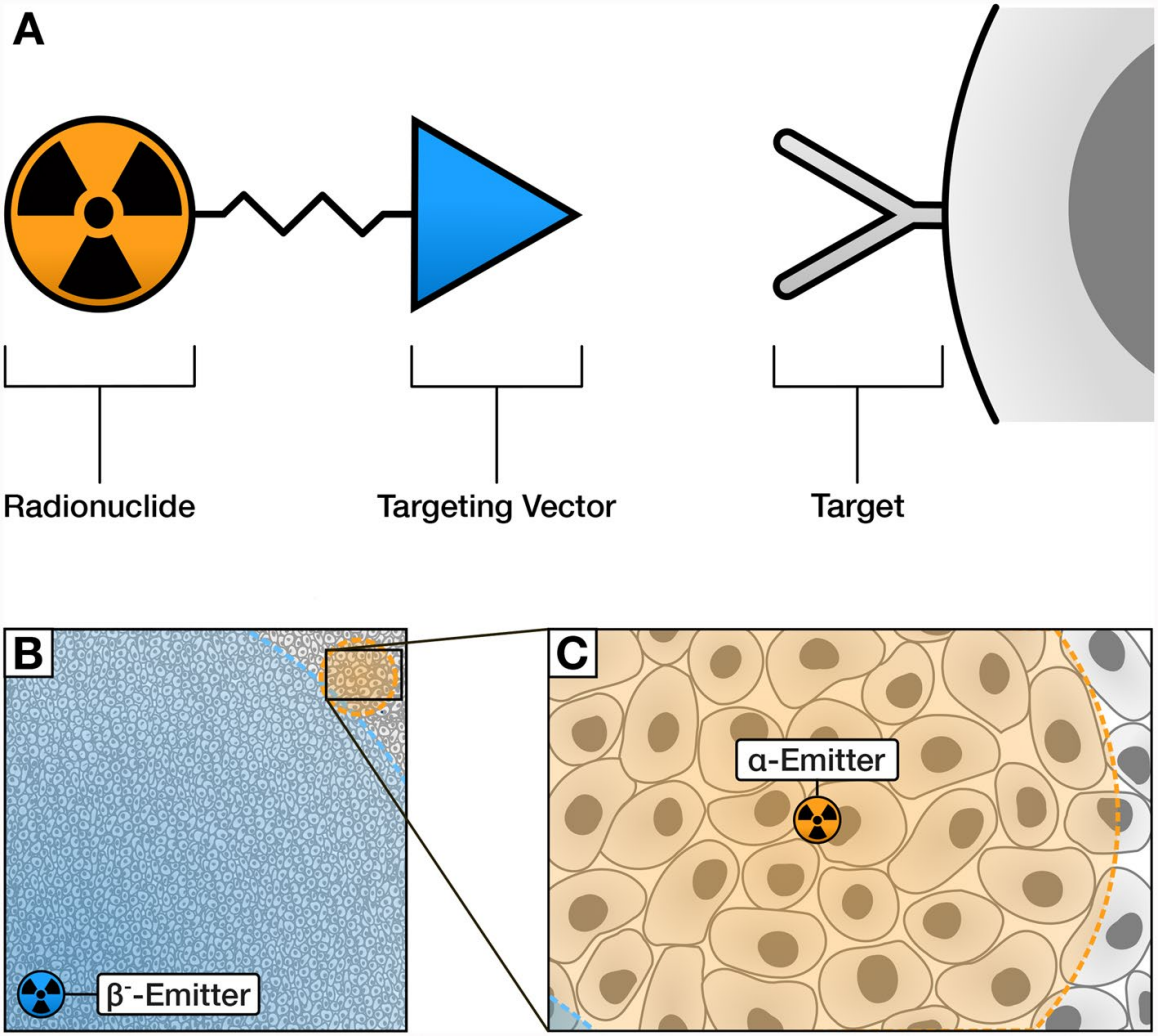
**A** Schematic of a radiopharmaceutical. **B** Relative range of β^−^ and **C** α particles in tissue

Targeted radionuclide therapy (TRT) is emerging as a safe and effective modality of cancer treatment (Sgouros et al. 2020; Stokke et al. 2022). In TRT, radionuclides which emit short-ranged ionising radiation (e.g. α or β^−^ particles) are selectively delivered to tumours through the use of a vector which binds specifically to cancerous cells, as depicted in Fig. 1a. The resulting radiopharmaceutical can be administered systemically, making it possible to treat disseminated and metastatic diseases that are otherwise challenging to manage with conventional radiotherapies. Unlike with chemotherapy, side effects are reduced as the targeted and short ranged radiation localises the cytotoxic effect to tumour, thus minimising harm to surrounding healthy tissue (Gill et al. 2017). Additionally, the success of TRT is far less dependent on complex cellular signalling and metabolic pathways, which are a substantial barrier to the development of typical targeted cancer therapies (Lin et al. 2019).

Radionuclides used for TRT can be classified depending on the type of radiation they emit, with most emitting β^−^ particles (electrons) or α particles (helium nuclei). Though most research has concerned the use of β^−^ emitters, radionuclides which emit α particles are particularly well-suited for use in TRT. Compared to β^−^ particles, α particles have over 1000 times the linear energy transfer, allowing them to generate highly cytotoxic DNA damage to which no cellular resistance mechanism is known (Sgouros et al. 2010; Suominen et al. 2017). Only a few α particles traversing the nucleus of a cell are sufficient to kill it, in contrast to the many thousands of β^−^ particles required to produce the same effect (Hassfjell 2000; Humm and Cobb 1990). α particles are effective against hypoxic tumours resistant to other forms of radiation, since their cytotoxicity is largely independent of the generation of reactive oxygen species (Hall and Giaccia 2006). This damage is also more localised, as their effective range in tissue is on the order of micrometres rather than millimetres (Fig. 1b) (Pouget and Constanzo 2021; Zalutsky and Bigner 1996). These properties make α-emitting nuclides ideal for the treatment of micrometastases, treatment resistant tumours, and haematological disease, whilst limiting damage to surrounding tissue.

To date, ^223^Ra is the only α-emitting nuclide that has been approved for clinical use. ^223^Ra is a bone-seeking agent on account of its chemical similarity to Ca^2+^, and is administered as a dichloride salt in the treatment of skeletal cancers (Deshayes et al. 2017; Suominen et al. 2017). However, its application has not extended to the treatment of other cancers, as attempts to conjugate ^223^Ra to various cancer-targeting vectors have proven unsuccessful due a lack of suitable chelating agents (Gott et al. 2016). Alternative radionuclides must thus be investigated to expand the scope of α-based therapies (Eychenne et al. 2021). ^212^Pb is one such radionuclide, which continues to garner interest for use in TRT (Kokov et al. 2022). While ^212^Pb itself is a β^−^ emitter with a 10.6 h half-life, it decays into the α-emitter ^212^Bi. ^212^Bi has long been investigated for therapy, though its short 60.5 min half-life greatly limits its application (Hassfjell and Brechbiel 2001). This can be overcome by instead using ^212^Pb as an in vivo generator of ^212^Bi, as its longer half-life effectively prolongs that of ^212^Bi. This approach has the additional advantage that the effective dose per unit of activity administered is approximately 50 times greater than with ^212^Bi alone (Milenic et al. 2008).

^212^Pb offers a several advantages over other α-emitters (Eychenne et al. 2021). It can be readily obtained from operationally simple radionuclide generators (Kokov et al. 2022). Its 10.6 h half-life is sufficient to permit its preparation and administration, though short enough to minimise toxicity from prolonged radiation exposure. Numerous suitable chelators are available for ^212^Pb, permitting straightforward and stable conjugation to a diverse range of targeting vectors (Grieve and Paterson 2022). Additionally, ^212^Pb is elementally matched to the diagnostic nuclide ^203^Pb, which can be imaged via single-photon emission computed tomography (SPECT) to accurately predict its biodistribution and dosimetry (vide infra). After decades of research in its production, chelation chemistry, and tumour targeting strategies, significant commercial investment has allowed ^212^Pb to begin entering the clinic. (Hassfjell and Brechbiel 2001; Jang et al. 2023). The challenge now is to realise this potential, and translate ^212^Pb towards a new class of safe and effective cancer therapies. This review discusses the therapeutic development of ^212^Pb, and highlights some of the key challenges remaining towards its clinical adoption.

## Main text

### Production of ^212^Pb

**Fig. 2:**
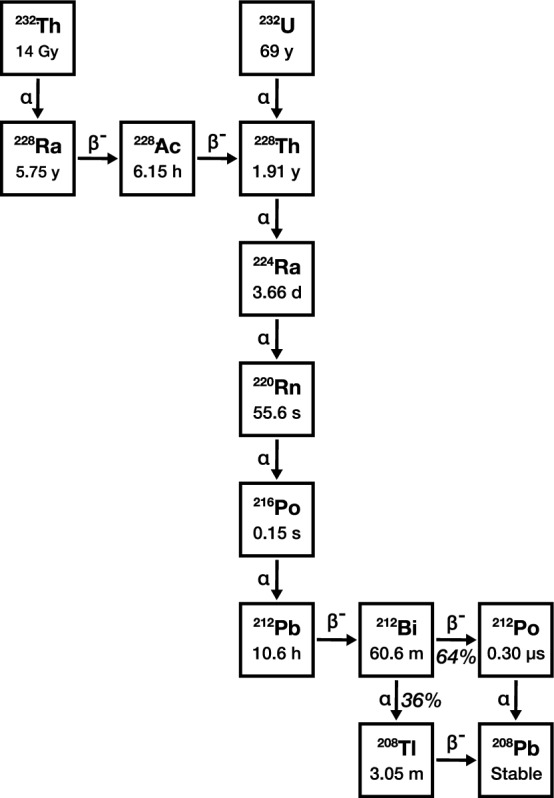
^232^Th & ^232^U decay chains. Half-lives and decay modes are shown, as are probabilities where the chain branches

As a member of the thorium series, ^212^Pb can be isolated from natural thorium deposits as the daughter of primordial ^232^Th (Cao et al. 2024; Chen et al. 2022; Narbutt and Bilewicz 1998). For nuclear medicine applications however, ^212^Pb is almost universally produced from generators based on the decay of ^228^Th (Fig. 2) (Kokov et al. 2022). Numerous methods can be used to acquire ^228^Th, which have been discussed in a recent review (Radchenko et al. 2021). ^228^Th can be obtained from the decay of ^228^Ra isolated from naturally occurring ^232^Th, though this method is limited by the sheer quantity of ^232^Th that must be processed to obtain appreciable amounts of ^228^Th. ^228^Th can instead be isolated as the daughter of ^232^U, a side product of the thorium cycle, and a component of legacy nuclear stockpiles (Humphrey and Khandaker 2018; Makvandi et al. 2018). ^226^Ra can also be used to produce ^228^Th in via neutron capture and subsequent β^−^ decay (Qiu et al. 2023). Finally, ^228^Th has also been obtained as a by-product of ^232^Th proton spallation used to produce ^225^Ac (McNeil et al. 2021). While the availability of ^228^Th has historically been limited, it can now be readily procured from numerous governmental and commercial organisations (Hassfjell 2001; Qiu et al. 2023; Zimmermann 2024).

There are a number of methods used to generate ^212^Pb for therapeutic purposes (Kokov et al. 2022). One approach is to adsorb ^228^Th onto some chromatographic material, which is eluted periodically to give ^212^Pb. In an early generator design, ^228^Th was adsorbed to a solid Na_2_TiO_3_ support, from which ^220^Rn was periodically eluted (Zucchini and Friedman 1982). After being left to decay, ^212^Pb could be isolated from the eluant via subsequent ion-exchange chromatography. Though these ^228^Th-based generators can theoretically be used for years, radiolytic damage of the generator over time decreases the yield and purity of isolated ^212^Pb, and can present a significant radiation hazard on larger scales (Atcher et al. 1988). To overcome this, ^228^Th can instead be kept in solution during daughter ingrowth, thereby reducing radiolytic damage to the chromatographic material (McAlister and Horwitz 2011; McNeil et al. 2023, 2021). This solution can then be loaded onto a metal selective resin to isolate ^212^Pb, while ^228^Th is returned to solution. By utilising a series of resins in tandem, this method can also allow the simultaneous isolation of ^224^Ra (McAlister and Horwitz 2011).

An alternative to the ^228^Th based generators it to instead use ^224^Ra as the parent nuclide for ^212^Pb generation. ^224^Ra is first chromatographically separated from ^228^Th or ^232^U, then adsorbed onto a cation exchange resin which functions as the ^212^Pb generator (Atcher et al. 1988; Baidoo et al. 2013; Li et al. 2017; Pruszyński et al. 2021). ^212^Pb can also be produced from ^224^Ra via the aforementioned liquid generator approach (Horwitz and Bond 2003; McAlister and Horwitz 2011). The key advantage of the ^224^Ra based generator is the relatively short half-life of ^224^Ra, which reduces long-term radiolytic damage to the generator material, and reduces handling of long lived ^228^Th. Indeed these ^224^Ra-based generators can now be procured commercially to avoid handling of ^228^Th entirely (Kokov et al. 2022; Zimmermann 2024). Of course, the short half-life of ^224^Ra is also the main limitation of this approach, as ^224^Ra based generators must be regenerated or replaced every 1–2 weeks.

An alternative generator strategy relies on the fact that gaseous ^220^Rn can be readily separated from a ^228^Th or ^224^Ra source, which is then allowed to decay to ^212^Pb. In practice, this can be achieved by simply confining the parent nuclide in an airtight container to collect emanated ^220^Rn, which subsequently deposits ^212^Pb on the container walls. The ^212^Pb can then be isolated in high purity by washing the container. This approach greatly simplifies ^212^Pb production, as it does not require chromatographic purification steps or frequent handling of the parent nuclide, and can be operational for many years when using ^228^Th as the parent. An early example of such a generator utilised a chamber containing ^228^Th doped barium stearate, selected on account of its high emanating coefficient for radon, and was able to reliably isolate high purity ^212^Pb (Hassfjell and Hoff 1994). Similar generator designs continue to be used for preclinical studies, on account of their operational simplicity (Artyukhov et al. 2022; Li et al. 2023c; Napoli et al. 2020). However, this approach is generally unsuitable for producing larger activities of ^212^Pb, as the collection process is challenging to automate, and radiolysis of the source material decreases yields over time. To overcome this, alternative designs generally separate the source from the collection chamber, with a stream of air carrying emanated ^220^Ra from the source to the collection chamber where it subsequently decays (Boldyrev et al. 2018, 2012; Hassfjell 2001). These designs are far easier to automate, and are less prone to radiolytic damage. While emanation based generators typically exhibit poorer yields than chromatographic designs, their advantages have attracted interest from a number commercial entities aiming to upscale production for clinical use (Zimmermann 2024).

While the above generator strategies aim to separate ^212^Pb from its longer-lived parent nuclides, solutions of ^224^Ra can be used directly for ^212^Pb radiolabelling (Stenberg et al. 2020a, b; Westrøm et al. 2017). In this approach, a solution of ^224^Ra is left to decay until it reaches equilibrium with its daughters, then the compound to be radiolabelled is added. Since the chemistry of Ra^2+^ and Pb^2+^ differ greatly, a compound bearing a suitable chelator can be selectively labelled with ^212^Pb in situ. A final chromatographic step then removes the ^224^Ra to give the ^212^Pb-labeled conjugate. While the process is faster and less laborious than conventional generators, the product may be still contaminated with residual ^224^Ra. Thus, further research is required to determine the clinical viability of this approach.

A challenge of ^212^Pb production is the hazard posed by the 2.6 MeV γ-emission of its ^208^Tl daughter. Due to its high energy, it can be difficult to provide adequate shielding to protect personnel, especially when high activities of ^212^Pb are required. Fortunately, the half-life of ^212^Pb is sufficient such that it can be delivered to treatment sites from a dedicated facility where the ^212^Pb-labeled radiopharmaceutical is produced. In this way, adequate shielding of the high activity generators can be more easily achieved than if ^212^Pb is produced in the clinic. Additionally, the relative simplicity of ^212^Pb generators permit automation of the entire ^212^Pb production and radiolabelling process, which serves to further minimise exposure to personnel (Li et al. 2017; Pretze et al. 2024).

### Conjugation strategies

**Fig. 3:**
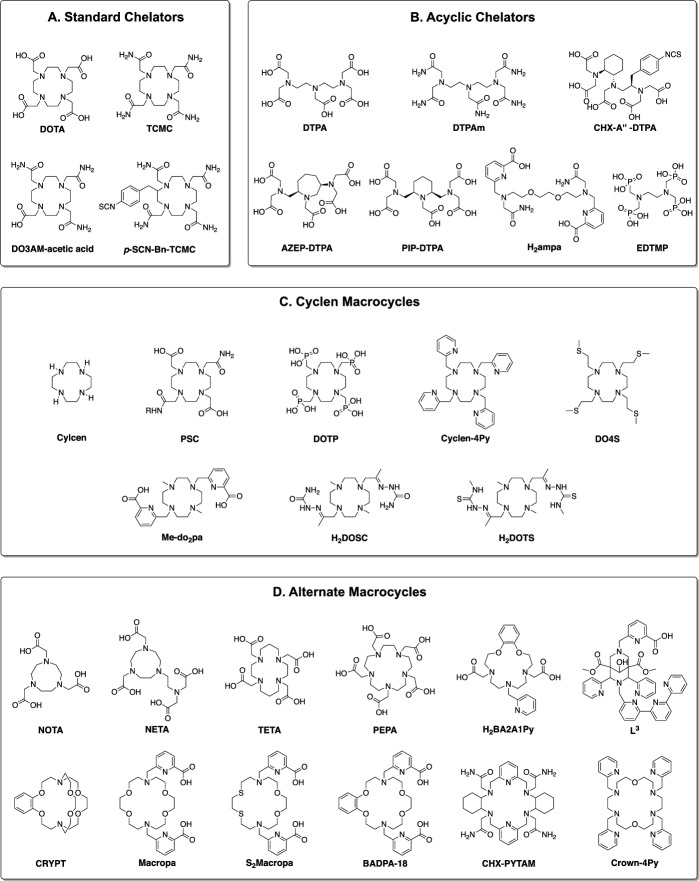
Selected chelators investigated for the ^212^Pb/^212^Bi pair. **A** Standard ^212^Pb Chelators. **B** Acyclic chelators. **C** Chelators incorporating the cyclen macrocycle. **D** Chelators incorporating alternative macrocycles

The therapeutic use of ^212^Pb necessitates sequestration of both ^212^Pb and ^212^Bi, as these nuclides naturally accumulate in key organs and produce toxicity (Durbin 1960; Milenic et al. 2015b; Milenic et al. 2001). Once sequestered, the radionuclide must also be conjugated to a targeting vector to facilitate tumour localisation and action. This is typically achieved using a ‘bifunctional’ chelator; containing both a metal binding moiety, and a reactive functional group to enable conjugation (Okoye et al. 2019; Sarko et al. 2012). Such a chelator must form biologically and kinetically inert complexes with ^212^Pb and ^212^Bi to prevent their release in vivo. Radiolabelling should be rapid, high yielding, and ideally occur under mild conditions. Following these criteria, numerous chelators have been investigated for ^212^Pb based TRT, as depicted in Fig. 3 and discussed further below.

#### Standard chelators

**Fig. 4:**
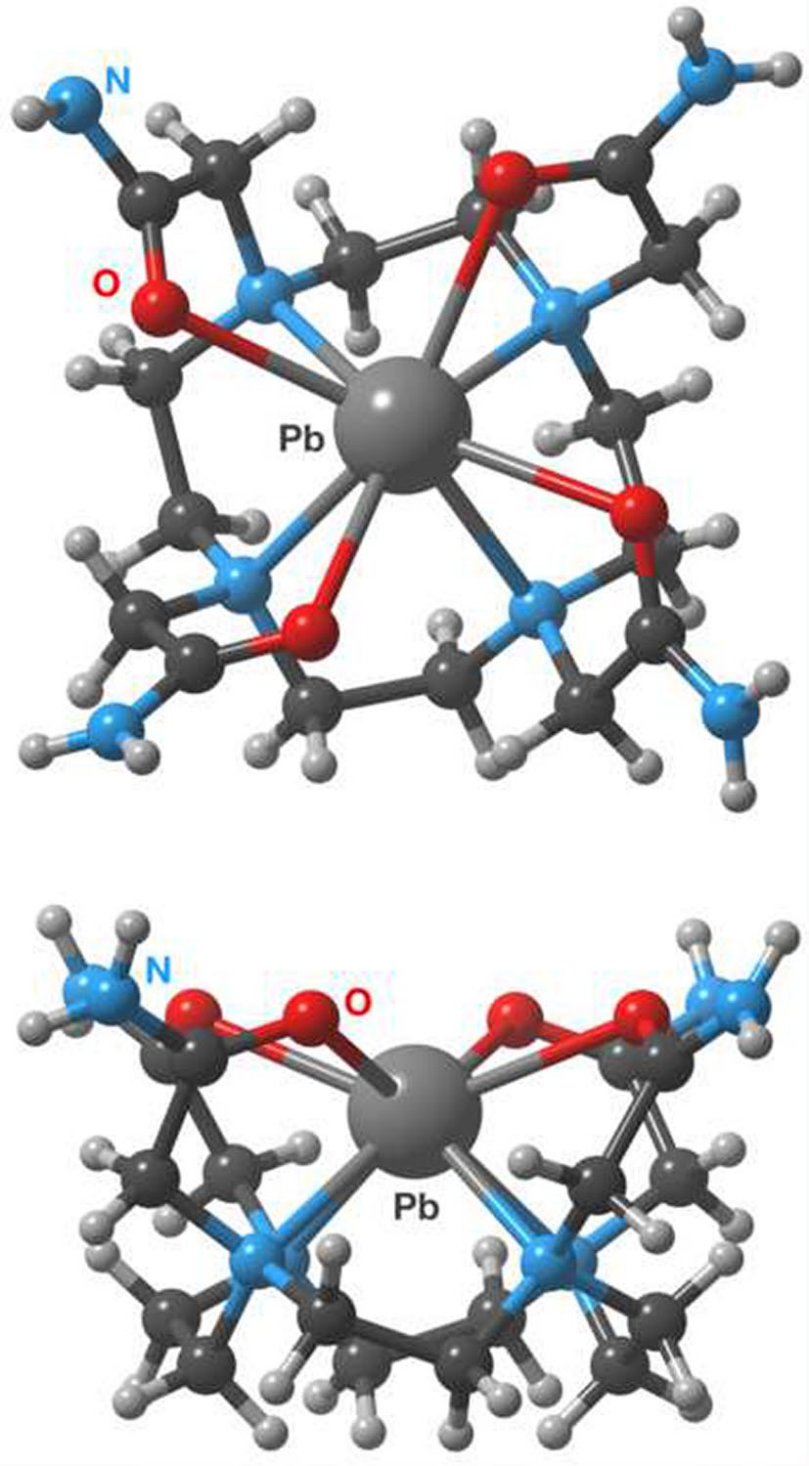
Crystal structure of the Pb-TCMC complex from a top down and side view

*N-*Functionalised cyclen derivatives are primarily used for ^212^Pb chelation, particularly 1,4,7,10-tetraazacyclododecane-1,4,7,10-tetraacetic acid (DOTA) and its tetra-amide derivative 1,4,7,10-tetraazacyclododecane-1,4,7,10-tetraacetic amide (TCMC a.k.a. DOTAM), depicted in Fig. 3a (Kokov et al. 2022). These chelators form analogous octadentate complexes with Pb^2+^ and Bi^3+^, coordinating via the cyclen nitrogens, and carboxylate or carboxamide oxygens respectively (Fig. 4) (Cuenot et al. 2008; Nugent et al. 2015). These complexes are thermodynamically and kinetically stable, and are highly inert in vivo over the half-life of ^212^Pb (Grieve and Paterson 2022; Pippin et al. 1995). However, Pb-DOTA complexes are prone to demetallation under acidic conditions (pH < 3.5), such as those encountered during cellular internalisation (Chappell et al. 2000). This limitation is not suffered by TCMC, which can also be efficiently radiolabelled under milder conditions than DOTA. For these reasons, TCMC has emerged as the standard chelator for ^212^Pb.

TCMC is typically conjugated to targeting vectors using one of two bifunctional derivatives. The first is *p*-SCN-Bn-TCMC, where is the cyclen backbone is functionalised with an isothiocyanate to allow conjugation to amines via the formation of a thiourea (Chappell et al. 2000). The conjugation reaction occurs under aqueous alkaline conditions, and is commonly employed to conjugate TCMC to antibody targeting vectors. Alternatively, DO3AM-acetic acid contains a carboxylate pendent arm which also permits conjugation to amine containing targeting vectors via amidation (Jurek et al. 2018). Since this reaction is compatible with typical solid phase peptide synthesis techniques, DO3AM-acetic acid is often used to introduce TCMC to peptide and small molecule targeting vectors. It is however unclear as to what extent conjugation via the carboxamide affects the in vivo stability of the resulting Pb-TCMC complex compared to the *C*-functionalised *p*-SCN-Bn-TCMC. Either bifunctional derivative may be further modified to permit versatile conjugation to alternative functional groups, including carboxylic acids, thiols, azides, and alkynes (Cardinale et al. 2019; Price and Orvig 2014). They can also facilitate bioorthogonal ‘click’ reactions such as the strain-promoted azide–alkyne cycloaddition and inverse electron demand Diels–Alder reactions, which permit chemically benign conjugation to a targeting vector in vivo (Bauer et al. 2024, 2023; Shah et al. 2017).

Despite the ubiquity of TCMC, alternative chelators have also been investigated for the ^212^Pb/^212^Bi pair. This is for three main reasons. The first is to improve the efficiency of radiolabelling. Radiolabelling is typically the final step of a radiopharmaceutical preparation, and thus the entire radiopharmaceutical construct is exposed to the radiolabelling conditions. While milder than DOTA, TCMC still generally requires heating and an acidic environment for efficient radiolabelling, which may not be compatible with more sensitive targeting vectors (Yang et al. 2022). Efficient radiolabelling also typically requires a large excess of chelator; thus a more efficient chelator could permit a greater specific activity of the resulting radiopharmaceutical. The second is to alter the physical properties of final radiopharmaceutical to improve its biological activity. Chelators with differing charges, hydrophilicity, and lipophilicity are likely to affect the pharmacokinetics and distribution of the final radiopharmaceutical (Bauer et al. 2024; Lee et al. 2024; Li et al. 2023b; McNeil et al. 2024). With a larger library of suitable ^212^Pb chelators to choose from, the biological properties of a radiopharmaceutical could be tailored simply by altering the chelator employed. Finally, alternate chelators may be more effective at retaining ^212^Bi following the decay of ^212^Pb, as discussed later in this review.

#### Acyclic chelators

Acyclic chelators such as diethylenetriaminepentaacetic acid DTPA have been historically used in the treatment of acute lead poisoning (Fischer et al. 1998). These ligands generally exhibit greater rates of formation than their macrocyclic counterparts, though they are also more labile. While this lability precludes the use of DTPA in TRT, its derivatives have been investigated for use with the ^212^Pb/^212^Bi pair, which are depicted in Fig. 3b (Montavon et al. 2012). The amide derivative DTPAm displayed more rapid radiolabelling than TCMC, though its radiolabelling efficiency was lesser, and its kinetic stability was not evaluated (Ingham et al. 2021). Improvements in kinetic stability have been achieved by incorporating rigid groups into the DTPA backbone. Incorporation of *trans*-cyclohexyl and a benzyl isothiocyanate groups within the DTPA backbone gives the bifunctional derivative CHX-A″-DTPA, which effectively chelate a range of radionuclides including isotopes of bismuth. (Brechbiel and Gansow 1992; Brechbiel et al. 1996; Milenic et al. 2001; Milenic et al. 2002; Price and Orvig 2014). However, CHX-A″-DTPA has not seen use for the chelation of ^212^Pb. An alternate strategy was employed by incorporating an azepane or piperidine ring to give AZEP-DTPA or PIP-DTPA respectively (Chong et al. 2006). While AZEP-DTPA was ineffective at retaining Pb^2+^ or Bi^3+^ in vivo, PIP-DTPA complexes were stable, warranting further investigation. Alternate classes of chelators have also been investigated, such as H_2_ampa which displays rapid and mild radiolabelling with Pb^2+^ and Bi^3+^, though its in vivo stability has not been evaluated (Ingham et al. 2022). Likewise, the phosphonate containing EDTMP has been investigated to facilitate bone targeting, though its ^212^Pb/^212^Bi complexes are unstable in vivo (Hassfjell et al. 1997, 1994). In general, acyclic chelators have generally exhibited poor radiolabelling, or have been too labile for clinical use with ^212^Pb (Bartoś et al. 2013; Ingham et al. 2021).

#### Macrocyclic cyclen-derived chelators

Like TCMC and DOTA, most alternate ^212^Pb/^212^Bi chelators investigated have also been derived from the cyclen macrocycle, as shown in Fig. 3c. Other than TCMC, Pb specific chelator (PSC) is the only such chelator to be used in clinical trials, which was designed specifically to reduce the net charge of its complexes (Li et al. 2023b; Michler et al. 2024). Pb^2+^ and Bi^3+^—TCMC complexes confer a net charge of + 2 and + 3 respectively to the radiopharmaceutical, and this positive charge may contribute to hepatic and renal retention (Bapst and Eberle 2017; Jones-Wilson et al. 1998). With its two carboxylate pendent arms, PSC reduces the net charge by 2, which may improve the biodistribution of some radiopharmaceuticals. However, PSC displays slower and less efficient radiolabelling than both TCMC and DOTA, and in some cases this reduction in net charge may be detrimental to the biodistribution of the resulting radiopharmaceutical (Saidi et al. 2025). With its chemical similarity to DOTA, PSC complexes may also have decreased acidic stability compared to TCMC, though this warrants further investigation.

DOTA derivatives bearing pendant arms besides carboxylate and carboxamides have also been proposed for ^212^Pb chelation. Phosphonate containing DOTP has been investigated as a bone targeting agent with ^212^Pb and ^212^Bi. It can be rapidly radiolabelled with Pb^2+^ and Bi^3+^, and its bismuth complex is kinetically and biologically stable (Hassfjell et al. 1997; Horváth et al. 2021). However, its ^212^Pb complex exhibits less stability in vivo, which has precluded further investigation (Bartoś et al. 2013). A series of DOTA derivatives substituting the carboxylates with pyridyl pendent arms have also been explored. Increasing the number of pyridyl groups increased the radiolabelling efficiency, with cyclen-4Py exhibiting Pb^2+^ labelling comparable to TCMC (McNeil et al. 2022, 2021). The stability of the resulting Pb^2+^ and Bi^3+^ complexes were also comparable to TCMC, warranting in vivo investigation (Wilson et al. 2015). Pyridinyl nitrogens are softer Lewis bases than carboxylate ions, and thus are thought to be more compatible with the borderline acids Pb^2+^ and Bi^3+^. Conversely, further increasing the softness of the donor group with sulphide groups as in DO4S had a deleterious effect on radiolabelling and complex stability compared to TCMC (Tosato et al. 2024). This underscores the importance of appropriately matching donor atoms to the metal ions.

An alternative chelator strategy is to utilise a cyclen derivative with two bidentate pendent arms. Me-do_2_pa is one such derivative, bearing two picolinate pendant arms (Lima et al. 2015; Lima et al. 2014). It can be rapidly labelled with Pb^2+^ and Bi^3+^ to form kinetically stable octadentate complexes, though they have not yet been evaluated in vivo. A semicarbazone derivative, H_2_DOSC displayed rapid and kinetically stable Pb^2+^ and Bi^3+^ complexation, though labelling was less efficient than with DOTA (Lange et al. 2020). An improved thiosemicarbazone derivative, H_2_DOTS, exhibited ^212^Pb radiolabelling comparable to TCMC, with excellent serum stability (Grieve et al. 2024). Its greater lipophilicity compared to TCMC was also demonstrated, suggesting it may be useful in modifying the pharmacokinetics once conjugated to a radiopharmaceutical.

#### Other macrocyclic chelators

While cyclen-derived ligands dominate ^212^Pb research, chelators derived from other macrocycles have also been studied, as shown in Fig. 3d. In contrast to the 12-membered cyclen, NOTA contains a smaller, 9-membered aza-crown ether core. Decreasing the size of the macrocycle in this way has a deleterious effect on Pb^3+^ binding, as the ligand is unable to accommodate the large size of the Pb^2+^ ion (Bartoś et al. 2013; Kumar et al. 1989). Pb^2+^ binding was improved by including additional donor atoms as in the NOTA derivative NETA, though in vivo stability remained poor (Chong et al. 2006). Using a larger, 14-membered aza-crown ether as in TETA also decreases binding affinity and complex stability compared to DOTA, which is thought to be due to the macrocyclic backbone adopting an unfavourable geometry (Dadachova et al. 1999; Kumar et al. 1989; Tosato et al. 2024). PEPA, with its 15 membered aza-crown macrocycle, is capable of binding to Pb^2+^ and Bi^3+^, though its Bi^3+^ complex exhibits poor in vivo stability (Dadachova et al. 1999; Garmestani et al. 2001). Chelators derived from other 15 membered aza-crown ethers, such as H_2_BA2A1Py, demonstrate rapid Pb^2+^ complexation and high stability in serum, though no in vivo data has been reported (Egorova et al. 2022). Bispidine chelators have also been investigated in vitro, with the nonadentate L^3^ displaying more efficient radiolabelling with ^212^Pb than TCMC at 40 °C. (Kopp et al. 2023).

The most efficacious non-cyclen derived ^212^Pb chelators have contained 18-membered aza-crown macrocycles. CRYPT, a [2,2,2] cryptand derivative, forms complexes with Pb^2+^ that exhibit high kinetic and thermodynamic stability, though greater heating, and longer reaction times than TCMC are required for efficient radiolabelling (McDonagh et al. 2021; McNeil et al. 2023). Nevertheless, bifunctional CRYPT derivatives are being investigated in vivo (McNeil et al. 2024). Macropa, originally developed for the chelation of lanthanides, forms highly thermodynamically stable complexes with Pb^2+^ and Bi^*3*+^ (Blei et al. 2023; Fiszbein et al. 2021). Additionally, macropa has been shown to surpass TCMC in radiolabelling rate and efficiency, and remains kinetically stable in vitro (Randhawa et al. 2024). Substitution of ether oxygens with sulphides was deleterious to radiolabelling, though radiolabelling with S_2_Macropa was still more favourable than TCMC. Addition of a benzyl group to the macropa backbone (BADPA-18) produced a chelator with a similar radiolabelling efficiency and kinetic stability as macropa, with quantitative Pb^2+^ radiolabelling observed within 2 min at ambient temperature (Zubenko et al. 2024). Further increasing the rigidity of the macrocyclic backbone as in CHX-PYTAM, produced Pb^2+^ complexes with a kinetic stability comparable to TCMC, though requiring harsher radiolabelling conditions (Harriswangler et al. 2024b). Notably, substituting the pendant arms of BADPA-18 or CHX-PYTAM with carboxylate groups negatively impacted Pb^2+^ complexation, as did substituting the macrocycle for a larger 21 membered aza-crown ether (Harriswangler et al. 2024a; Zubenko et al. 2024). Likewise, the pyridyl containing Crown-4Py exhibited worse Pb^2+^ labelling than either TCMC or cyclen-4Py, further highlighting the importance of matching both the donor atoms and macrocycle size to the metal ions for efficient chelation (McNeil et al. 2022).

#### Alternative carriers

**Fig. 5:**
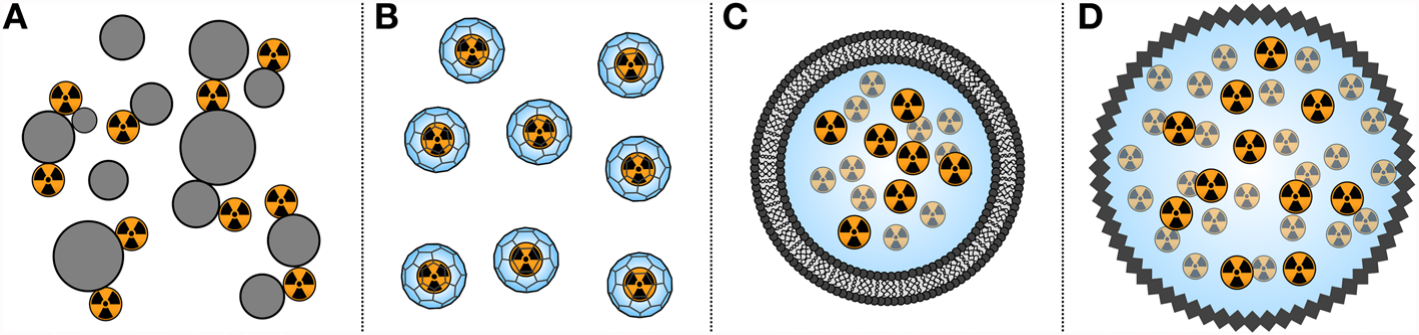
Alternative carriers investigated for use with ^212^Pb. **A** Colloids. **B** Fullerenes. **C** Liposomes. **D** Nanoparticles

Rather than relying on chelation chemistry, ^212^Pb can instead be sequestered by larger constructs, as depicted in Fig. 5. The earliest reported therapeutic studies of ^212^Pb employed sulphur and iron colloids, which were effective at retaining ^212^Pb in vivo (Rotmensch et al. 1989a, b). A similar effect has been reported with ^212^Pb adsorbed CaCO_*3*_ microparticles (Li et al. 2021b). However, these strategies do not permit conjugation of a targeting vector to enable tumour localisation, thus limiting their application to the treatment of intracavitary cancers.

An alternative strategy is to encapsulate ^212^Pb within a nanoconstruct that can be functionalised with targeting vectors at its surface. C_60_ fullerenes have been used to encapsulate radiometals and can be derivatised to facilitate target vector conjugation (Ashcroft et al. 2006; Shultz et al. 2010). A study on endohedral ^212^Pb containing fullerenes demonstrated that such complexes are stable in vivo, though the radiolabelling efficiency was less than 1% (Diener et al. 2007). Liposomes have also been studied, which are attractive carrier options since they can be extensively modified to tailor their biodistribution and pharmacokinetic profiles (Large et al. 2021, 2019). Liposomes incorporating ^212^Pb exhibit high in vitro stability and can be radiolabelled in high yields, though in vivo studies are limited (Du et al. 2011; Pikul et al. 1987; Rosenow et al. 1983). More recently, silver telluride nanoparticles have been used to encapsulate ^212^Pb, with the resulting constructs remaining highly stable in vitro (Wang et al. 2024). While nanoconstructs are increasingly finding medicinal applications, the use of such constructs with ^212^Pb remains limited (Majkowska-Pilip et al. 2020; Mitchell et al. 2021). Future studies are required in order to determine if they are viable alternatives to chelation chemistry, given the restrictions imposed by the half-life of ^212^Pb.

### Dissociation of ^212^Bi

The nuclear recoil effect describes the kinetic energy imparted to a nucleus following radioactive decay. In the case of high energy emissions, particularly α-particles, this recoil energy can be sufficient to break chemical bonds. Complex dissociation is thus expected to occur following α-decay of ^212^Bi or ^212^Po, though as ^208^Tl is a short-lived β^−^ emitter and ^208^Pb is stable, this is not a major concern (Orcutt et al. 2022). Of greater issue is the dissociation of ^212^Bi following the decay of ^212^Pb. Studies of [^212^Pb]Pb-DOTA and [^212^Pb]Pb-TCMC complexes suggest that around 30% ^212^Bi is released, despite the recoil energy of the β^−^ decay being theoretically insufficient to break metal–ligand bonds (Mirzadeh et al. 1993; Westrøm et al. 2017). Instead, this has been attributed to the internal conversion which occurs following a percentage of ^212^Pb decays (Mirzadeh et al. 1993). The resulting ^212^Bi ions are produced in electronically excited states, and the reorganisation of valence electrons that follows is thought to destabilise the complex. Clinically, the released ^212^Bi accumulates in the kidneys and elicits dose limiting toxicity (Banerjee et al. 2020; Zidenberg-Cherr et al. 1987). As this represents a limitation to ^212^Pb therapies, several methods are being developed to mitigate the toxicity of the released ^212^Bi (De Kruijff et al. 2015).

One solution is to confine disassociated ^212^Bi to the site of the tumour through locoregional injection. For instance, intra-peritoneal (i.p.) administration is effective at preventing renal accumulation of ^212^Bi, as Bi^3+^ does not readily redistribute from the peritoneal cavity (Kasten et al. 2017; Meredith et al. 2014b; Rotmensch et al. 1997). Alternatively, by targeting receptors which internalise upon ligand binding, ‘free’ ^212^Bi can be sequestered intracellularly within the tumour (Li et al. 2023b; Miao et al. 2005; Milenic et al. 2005; Sabbioni et al. 2021). The later approach necessitates rapid clearance and tumour uptake to minimise toxicity from ^212^Bi lost during circulation, thus radiopharmaceuticals with slower pharmacokinetics are likely to see less benefit (Boudousq et al. 2013). Either approach also limits the types of cancers that can be treated, respectively requiring localised disease, or expression of internalising receptor types.

Another strategy is to increase the rate at which the ^212^Bi is excreted. Studies of ^225^Ac-labeled radiopharmaceuticals have demonstrated that co-administration of diuretics and heavy metal chelating agents can prevent kidney toxicity from the subsequent release of its ^213^Bi daughter (Jaggi et al. 2006; Singh Jaggi et al. 2005). Co-administration of dithiol chelator 2,3-dimercapto-1-propanesulfonic acid (DMPS) prevents renal accumulation of systemically administered Bi^3+^, though the use of DMPS alongside an internalising peptide-targeted ^212^Pb radiopharmaceutical was deemed unnecessary in an animal model (Jones et al. 1996; Miao and Quinn 2008). Likewise, in a clinical study of i.p. administered [^212^Pb]Pb-trastuzumab, a diuretic regime was stopped early as no evidence of renal toxicity was apparent (Meredith et al. 2014a; Meredith et al. 2018). In either case, disassociated ^212^Bi appeared to be sufficiently confined to the tumour, such that renal accumulation did not occur. In therapies where dissociated ^212^Bi is not as well sequestered though, this strategy may still prove useful at reducing renal toxicity.

^212^Bi may also be retained though the use of alternative chelating agents. One proposed approach is to use a chelator with a high affinity for Bi^3+^, such that disassociated ^212^Bi readily reassociates. Such chelator must be able to rapidly bind to ^212^Bi in a highly dilute environment, since radiopharmaceuticals are administered at nanomolar biological concentrations (Galbiati et al. 2024). DOTP has been investigated for this purpose, though it was only effective at retaining ^212^Bi at concentrations above 1 mM (Bartoś et al. 2013). A large excess of unlabelled ligand would be required to reach these concentrations during therapy, which would hinder tumour localisation of the radiopharmaceutical by competitively binding to the target site. It has alternatively been suggested that chelators with extended, delocalised pi-systems could prevent dissociation by rapidly neutralising the charge of the electronically excited daughter nuclide (Nath et al. 2001; Zeevaart et al. 2012). Porphyrins represent one such class of chelators, and thus have been investigated for this purpose in other in vivo generator systems. Like ^212^Pb, ^166^Dy is a β^−^ emitter proposed as a generator for its daughter ^166^Ho. While up to 70% of the daughter is released from the [^166^Dy]Dy-DOTA complex following internal conversion, this release was prevented entirely when using a porphyrin chelator instead (Salek et al. 2020; Zeevaart et al. 2012). Although lead and bismuth porphyrin complexes have been reported, they have not yet been investigated with the ^212^Pb/^212^Bi pair (Halime et al. 2009, 2007). Thus, further investigation into porphyrins and other highly conjugated chelators may prove effective at retaining ^212^Bi.

Dissociation of ^212^Bi may also be prevented entirely using nanoconstructs which confine ^212^Pb and its daughters within. With other radionuclides, a range of constructs have been effective at retaining otherwise lost daughter nuclides (De Kruijff et al. 2015; Majkowska-Pilip et al. 2020; Mdanda et al. 2023; Silindir-Gunay et al. 2020). Accordingly, studies of ^212^Pb-labled liposomes and nanoparticles have demonstrated their efficacy in retaining ^212^Bi (Du et al. 2011; Henriksen et al. 2003; Pikul et al. 1987; Wang et al. 2024). On the other hand, ^212^Pb labelled C_60_ fullerenes were ineffective at retaining ^212^Bi, despite the stability of the endohedral ^212^Pb construct (Diener et al. 2007). Nevertheless, nanoconstructs represent a promising strategy to retain ^212^Bi, and additional research into their use with ^212^Pb is required to realise this potential.

### Targeting strategies and applications

The efficacy of TRT depends on the ability to differentiate between healthy and cancerous cells to minimise side effects. ^212^Pb does not naturally accumulate in tumours, rather it is absorbed primarily by the bone, while its ^212^Bi daughter is retained by the kidneys (Durbin 1960). Thus, a strategy to facilitate tumour localisation must be employed. This is typically achieved using a targeting vector—a molecule that binds with high specificity to a biological target (e.g., a receptor, enzyme, metabolite) produced in abundance by cancerous cells but not healthy tissue (Sgouros et al. 2020; Vermeulen et al. 2019). This target should ideally be expressed by metastasised cells in addition to the primary tumour and be located extracellularly such that it is accessible to the circulating radiopharmaceutical. It can also be advantageous for the target to internalise upon ligand binding, such that ^212^Pb is confined intracellularly and retained at the tumour as previously discussed. Internalising, cell surface receptors thus represent the most common targets for TRT. Importantly, the choice of targeting vector generally determines a radiopharmaceutical’s pharmacokinetic properties, with small molecule, peptide, and IgG antibody-based targeting vectors primarily studied for TRT (Sgouros et al. 2020).

Due to their smaller size, small molecule and peptide targeted radiopharmaceuticals generally exhibit more rapid tumour uptake and clearance than those targeted by antibodies, and can more easily permeate tissue in the treatment of solid cancers. (Mould and Green 2010; Sgouros et al. 2020). However, they also typically have shorter tumour retention times that can dimmish their effectiveness, and peptides are particularly prone to enzymatic degradation (Cooper et al. 2021). Furthermore, small molecules and peptides are limited in types of biological structures they can target, generally requiring well defined enzyme or receptor binding pockets. Antibodies on the other hand can bind to biological structures that are not easily targeted by small molecules, greatly extending the range of cancers that can be treated (Ministro et al. 2020). Their long tumour retention time allows a sustained dose to be administered, though their long circulating half-life and slower clearance can lead to off target irradiation. It can also be challenging for antibodies to permeate tissues and target non-haematological tumours on account of their large size (Mould and Green 2010; Sgouros et al. 2020). Thus, the careful selection of an appropriate targeting vector is vital to the success of a radiopharmaceutical.

#### Untargeted

A targeting vector is not strictly necessary if ^212^Pb can be confined to the tumour though some other mechanism. One such strategy is to treat peritoneal cancer by i.p. administration. Early studies investigating the use of ^212^Pb-labelled colloids demonstrated dose dependent survival in murine models of ovarian carcinoma, with minimal redistribution outside of the peritoneal cavity (Rotmensch et al. 1989a, b). A more recent study also demonstrated efficacy against murine ovarian cancer with ^212^Pb adsorbed CaCO_3_ microparticles, though the ^212^PbCl_2_ control was equally efficacious (Li et al. 2021b). The microparticles were however effective at confining ^212^Pb within the peritoneal cavity, as evident by minimal organ redistribution compared to the control. While significant toxicity was not noted in these pilot studies, off target radiation within the peritoneal cavity may preclude this approach from entering the clinic.

Despite not localising to the tumour, some level of therapeutic efficacy has also been observed preclinically with ^212^Pb-labeled i.p. and i.v. administered non-specific antibodies (Kasten et al. 2017; Kasten et al. 2018b; Milenic et al. 2007), untargeted small molecules (Banerjee et al. 2020), and even ^212^PbCl_2_ itself (Li et al. 2021b). This effect has also been reported with other untargeted α-emitting nuclides, and it has been proposed to be the result of a systemic anti-tumour immune response which arises following treatment (Milenic et al. 2004; Pouget and Constanzo 2021; Roncali et al. 2024). Indeed, it is well documented that radiotherapy can induce tumour cell death outside of the irradiated area, a phenomenon termed the ‘abscopal effect’ (Demaria and Formenti 2020; Siva et al. 2015). Radiotherapy has also been shown to potentiate the effects of immunotherapy, and to this end numerous TRTs are being investigated in combination with immunotherapy regimes (Kerr et al. 2022; Sun et al. 2023). This potentiation has also been observed alongside untargeted, low dose, whole-body irradiation (Dong et al. 2025; Jing et al. 2014; Nowosielska et al. 2021). Untargeted ^212^Pb based therapies may thus have some therapeutic value at low doses, especially in combination with existing treatment modalities, though this remains a poorly studied area of research.

#### Small molecule and peptide targeted

One method to facilitate tumour localisation is to complex ^212^Pb with a chelator that naturally accumulates in the target tissue. Phosphonates exhibit an affinity for bone tissue, and thus phosphonate containing chelators can effectively localise radiometals to the bone (Lange et al. 2016). [^153^Sm]Sm-lexidronam is the most prominent example of this approach, a phosphonate containing radiopharmaceutical prescribed for the management of metastatic bone pain (Longo et al. 2013; Sartor 2004). In the case of ^212^Pb, chelation with EDTMP or DOTP (Fig. 3) effectively localises ^212^Pb to the bone (Hassfjell et al. 1997, 1994). While preferential localisation to sites of high bone turnover was demonstrated, efficacy studies against skeletal cancers have not been reported.

**Fig. 6:**
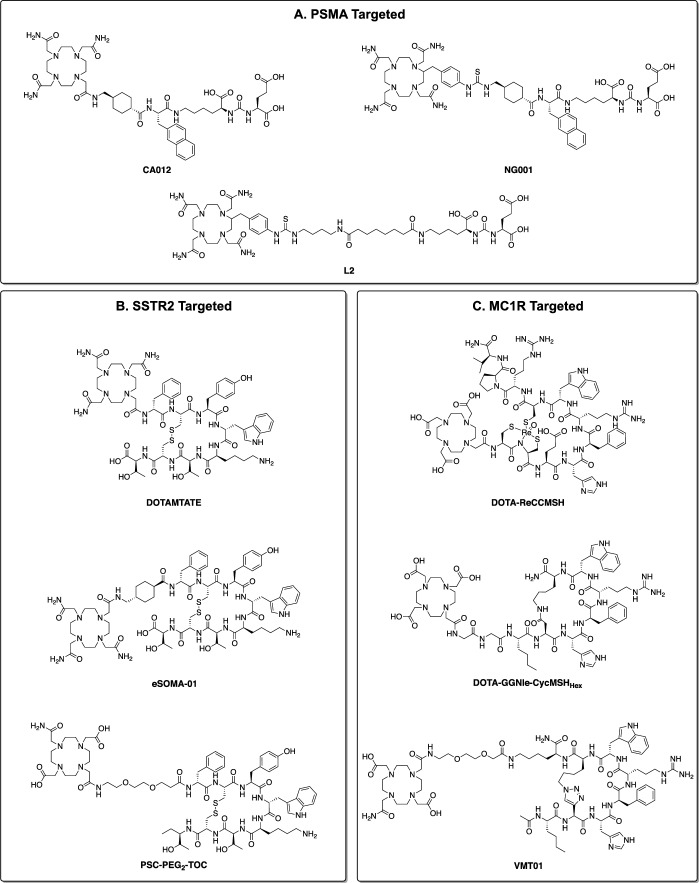
Selected clinically and preclinically investigated small molecule and peptidic ^212^Pb ligands which target: **A** prostate specific membrane antigen (PSMA); **B** somatostatin receptor subtype 2 (SSTR2); **C** melanocortin receptor 1 (MC1R)

More commonly, tumour targeting is achieved by conjugating ^212^Pb to a molecule that binds to a specific protein expressed by cancerous cells. Prostate specific membrane antigen (PSMA) is a transmembrane glycoprotein highly overexpressed by prostate cancers, and can be effectively targeted with a Glu-urea-Lys binding motif, a mimic of its natural substrate (Afshar-Oromieh et al. 2016; Maurer et al. 2016; Ruigrok et al. 2019; Wang et al. 2022a). Its effectiveness as a target for TRT has been exemplified by the recent regulatory approval of the PSMA targeting β^−^ emitter, [^177^Lu]Lu-PSMA-617 (Fallah et al. 2023; Keam 2022). The feasibility of ^212^Pb-based PSMA therapy was first evaluated with [^212^Pb]Pb-CA012, the TCMC analogue of PSMA-617 (Fig. 6a) (dos Santos et al. 2019). Maurine and human biodistribution studies confirmed a high tumour uptake, and rapid kidney clearance. Dosimetry estimates identified the kidneys and salivary glands as dose limiting organs, with 150 MBq estimated as the maximum tolerated dose of [^212^Pb]Pb-CA012. In another study, ^212^Pb-L2 exhibited dose dependent tumour inhibition, and a greater survival benefit than [^177^Lu]Lu-PSMA-617 in a metastatic disease model, though renal toxicity was observed at therapeutic doses (Banerjee et al. 2020). A series of preclinical studies have also been performed on the PSMA-617 derivative [^212^Pb]Pb-NG001 (Stenberg et al. 2020a; Stenberg et al. 2021). Dose dependent tumour inhibition and survival was demonstrated, and significant kidney toxicity was not observed at therapeutic doses, attributed to the rapid clearance, and high internalisation rate of [^212^Pb]Pb-NG001. Efficacy was also observed in animal models of both solid tumours, and micrometastases (Stenberg et al. 2022). In a subsequent phase 0 clinical trial, subtherapeutic doses (< 10 MBq) of [^212^Pb]Pb-NG001 displayed tumour localisation and a favourable biodistribution, warranting further clinical investigation (Berner et al. 2025). A phase I/IIa clinical trial is also underway with the PSMA targeting [^212^Pb]Pb-ADVC001 (ACTRN12622001378718), which has recently generated clinical ^212^Pb SPECT/CT images (Griffiths et al. 2024).

Somatostatin receptor subtype 2 (SSTR2) is a G-protein coupled receptor (GPCR) highly expressed by neuroendocrine tumours. The current standard of care for SSRT2 positive neuroendocrine tumours includes the β^−^ emitter [^177^Lu]Lu-DOTATATE, which contains the SSRT2 targeting peptide octreotide (Dierickx et al. 2020; Hennrich and Kopka 2019). [^212^Pb]Pb-DOTAMTATE, the ^212^Pb labelled TCMC derivative of DOTATE depicted in Fig. 6b, exhibited rapid tumour uptake and retention in an SSTR2 positive murine model (Stallons et al. 2019). While uptake was observed in the kidneys and pancreas, coadministration with kidney protective agents increased renal clearance. In an efficacy study, three 0.37 MBq doses of [^212^Pb]Pb-DOTAMTATE quadrupled median survival times in the same disease model (Stallons et al. 2019). This was further improved upon co-administration of the chemo-sensitising agent 5-fluorouracil, with 79% of animals tumour free after the 31-week study period. These results prompted a phase 1 dose escalation study, where [^212^Pb]Pb-DOTAMTATE was well tolerated in patients with only mild adverse effects reported and promising preliminary efficacy results reported (Delpassand et al. 2022). The 2.50 MBq/kg dose per cycle recommended by this trial is now being evaluated in a phase 2 clinical trial (NCT05153772)**.** Preclinical studies on [^212^Pb]Pb-eSOMA-01, a derivative of [^212^Pb]Pb-DOTAMTATE, demonstrated a superior biodistribution profile to [^212^Pb]Pb-DOTAMTATE in animal models, though efficacy studies have not been performed (Chapeau et al. 2023). A phase 1/IIa dose escalation study (NCT05636618) is also underway with [^212^Pb]Pb-PSC-PEG2-TOC, which utilises Tyr^3^octreotide as the SSRT2 targeting peptide (Michler et al. 2024). It was demonstrated that replacing TCMC with PSC, and including a polyethylene glycol linker improved receptor binding, tumour uptake, and renal clearance compared to DOTATOC (Lee et al. 2024; Li et al. 2023b). A survival benefit was demonstrated in a murine disease model, with a fractionated dose regime most efficacious as with [^212^Pb]Pb-DOTAMTATE.

Melanocortin receptor 1 (MC1R), a GPCR highly expressed by melanomas, has also been investigated as a target for ^212^Pb TRT. MC1R targeting vectors are typically derivatives of its native ligand, α-melanocyte stimulating hormone (α-MSH), a selection of which are shown in Fig. 6c (Castrucci et al. 1989; Hadley et al. 1989; Miao and Quinn 2021). Evaluation of the ruthenium cyclised α-MSH analogue [^212^Pb]Pb-DOTA-ReCCMSH demonstrated favourable biodistribution and a dose dependent survival benefit in an animal model, though renal toxicity was observed in higher doses, suggesting further optimisation of the peptide structure and dosing regimen was necessary (Miao et al. 2008, 2005). [^203^Pb]Pb-DOTA-GGNle-CycMSHHex, a lactam cyclised variant, exhibited a similar biodistribution profile, though it has not yet been evaluated for efficacy when labelled with ^212^Pb (Yang et al. 2019). Pharmacological upregulation of the MC1R receptor has been demonstrated to improve efficacy of MC1R targeted radiotherapies, as has co-administration with immune checkpoint inhibitors (Li et al. 2021a; Li et al. 2019). [^212^Pb]Pb-VMT01, a triazole cyclised α-MSH analogue bearing a PSC chelator, was efficacious in preclinical studies, and is currently being investigated in a phase I/IIa dose-escalation study in combination with the immune checkpoint inhibitor Nivolumab (NCT05655312) (Li et al. 2021a).

Several other peptide targeted ^212^Pb TRTs are being investigated clinically and preclinically. [^212^Pb]Pb-DOTAM-GRPR1 is a peptidic radiopharmaceutical targeting the gastrin releasing peptide receptor (GRPr), a GPCR expressed by a range of cancers including prostate, breast, pancreatic, and cervical (Patel et al. 2006; Saidi et al. 2024). In a murine prostate cancer model, treatment with [^212^Pb]Pb-DOTAM-GRPR1 more than doubled median survival times. The maximum tolerated dose surpassed the fatal dose of [^212^Pb]Pb-DOTAMTATE, with no toxicity observed with doses up to 1.67 MBq. [^212^Pb]Pb-DOTAM-GRPR1 is thus currently being investigated in a phase 1 dose finding study against GRPr expressing tumours (NCT05283330). Fibroblast activation protein (FAP) is a transmembrane serine protease overexpressed by a wide range of solid cancers (Fitzgerald and Weiner 2020; Shahvali et al. 2023). It can be targeted using small molecule and peptide inhibitors, and is increasingly being investigated as a TRT target (Privé et al. 2023). FAP targeted ^212^Pb radiopharmaceuticals have been reported, with one such set to begin a phase I/IIa study in 2025 (NCT06710756) after favourable preclinical results (Cagle et al. 2024; McNeil et al. 2024). Another phase I safety study is scheduled for [^212^Pb]Pb-Pentixather (NCT05557708), a peptidic radiopharmaceutical which targets chemokine receptor type 4 (CXCR4). CXCR4 is a cytokine GPCR, expressed by numerous cancers, particularly those that are metastatic (Sanchis-Pascual et al. 2024; Yu et al. 2023). Reported in a recent preprint, [^212^Pb]Pb-Pentixather increased survival in murine CXCR4 tumour models, though the risk of myelotoxicity clinically was noted due to the greater affinity of Pentixather for the human CXCR4 isoform expressed on hematopoietic stem cells (Christensen et al. 2024).

#### Antibody targeted

^212^Pb labelled TCMC-trastuzumab is perhaps the most studied ^212^Pb radiopharmaceutical agent. Trastuzumab is an IgG monoclonal antibody (mAb) which is internalised upon binding to the human epidermal growth factor receptor 2 (HER2), a membrane bound receptor overexpressed by a range of solid tumours (Oh and Bang 2020). I.p. injection of [^212^Pb]Pb-TCMC-trastuzumab dramatically increased survival times in mice bearing HER2 expressing xenografts, with subsequent injections further increasing survival (Milenic et al. 2005). Notably, the longer half-life of ^212^Pb was shown to be advantageous over the shorter lived ^212^Bi and ^213^Bi. Additional studies demonstrated improved survival times when combined with existing chemotherapies including gemcitabine, paclitaxel, and carboplatin (Milenic et al. 2013, 2007, 2008; Yong et al. 2013). These results prompted phase I clinical trials of i.p. [^212^Pb]Pb-trastuzumab in the treatment of ovarian and colon cancers, the first such trial of a ^212^Pb radiopharmaceutical, and the only of a ^212^Pb-antibody conjugate to date (Meredith et al. 2014a; Meredith et al. 2014b; Meredith et al. 2018). Doses up to 27 MBq/m^2^ were well tolerated by patients, produced little toxicity, and exhibited minimal distribution outside the peritoneal cavity. However, all patients failed to show a response at these doses, with all having disease progression within 8 months. [^212^Pb]Pb-trastuzumab may thus be efficacious at higher doses, and in combination with other therapies. However, despite the observed safety and tolerability of [^212^Pb]Pb-trastuzumab, no such phase II study was ever performed. Intravenous (i.v.) administration of [^212^Pb]Pb-trastuzumab has also been investigated pre-clinically in the treatment of HER2 expressing prostate cancer, exhibiting tumour uptake and a survival benefit without apparent systemic toxicity after a single 0.74 MBq injection (Schneider et al. 2013; Tan et al. 2012). However, a significant percentage of the injected dose was retained in the blood, spleen, kidneys, and liver.

This difference in systemic versus locoregional administration of a ^212^Pb-mAb has also been observed with antibodies targeting the B7 homolog 3 protein (B7-H3, a.k.a. CD276), a regulatory immune protein overexpressed by various cancer types (Zhou and Jin 2021). In two murine ovarian cancer models, i.p. administration of the B7-H3 targeting mAb construct [^212^Pb]Pb-376.99 prolonged survival compared to the untreated control groups (Kasten et al. 2017). However, at the same dose, median survival times were comparable between mice administered [^212^Pb]Pb-376.99, and a non-specific mAb conjugate [^212^Pb]Pb-F3-C25. Thus, the therapeutic efficacy of [^212^Pb]Pb-376.99 may not be due to its tumour targeting properties, but rather its ability to confine ^212^Pb to the peritoneal cavity. In a follow up study, i.v. administration of [^212^Pb]Pb-376.99 was investigated in a xenografic model of pancreatic ductal adenocarcinoma (Kasten et al. 2018a). [^212^Pb]Pb-376.99 exhibited greater tumour uptake than [^212^Pb]Pb-F3-C25, and produced a dose dependent reduction in tumour volume. Again however, only modest difference was observed in the tumour reducing ability of [^212^Pb]Pb-376.99 and [^212^Pb]Pb-F3-C25 administered at similar doses, and as with i.v. administered [^212^Pb]Pb-trastuzumab, a substantial amount of [^212^Pb]Pb-376.99 was retained in the blood, spleen, kidneys, and liver.

Efficacy treating peritoneal cancers has also been observed with other antibody targeted agents. ^212^Pb labelled mAbs targeting the epidermal growth factor receptor (EGFR a.k.a. HER1), cluster of differentiation 146 (CD146) and protein tyrosine kinase 7 (PTK7) have been investigated preclinically for colorectal cancer, mesothelioma, and ovarian cancer respectively (Lindland et al. 2024a; Lindland et al. 2024b; Milenic et al. 2015a; Milenic et al. 2017). All agents reduced tumour sizes and prolonged survival time compared to an untreated control, and a ^212^Pb conjugated non-specific antibody. ^212^Pb-mAb constructs thus continue to show promise in the treatment of peritoneal cancers.

Systemically administered ^212^Pb radioimmunotherapy have also been investigated, with varying levels of success. Vascular cell adhesion molecule 1 (VCAM-1) is a cell surface protein, involved with cell adhesion, metastasis, and angiogenesis (Kong et al. 2018; Sharma et al. 2017). Notably, VCAM-1 is upregulated during the early stages of brain metastasis (Soto et al. 2014). In an in silico brain metastases model, the most promising dosimetric outcomes were observed with a ^212^Pb labelled anti-VCAM-1 mAb (αVCAM) compared to [^177^Lu]Lu-αVCAM (Falzone et al. 2018). Efficacy was then confirmed in a murine metastatic breast cancer model (Corroyer-Dulmont et al. 2019). [^212^Pb]Pb-TCMC-αVCAM displayed a high uptake in metastatic brain tumours, sixfold greater than healthy brain tissue. Compared to whole brain radiotherapy, the standard of care for brain metastases, the number and volume of brain tumours markedly decreased after treatment, and survival times increased. No major toxicity was observed in the treated group beyond a decrease in body weight, thus warranting further study.

Promising preclinical results have also been reported in the treatment of lymphoid tumours. CD20 is a transmembrane protein expressed by B-cells, which is targeted by the clinically approved TRT β^−^ emitters [^90^Y]Y-ibritumomab tiuxetan and [^121^I]I-tositumomab in the treatment of non-Hodgkin’s lymphoma (Choi et al. 2020; Durando et al. 2024). [^212^Pb]Pb-rituximab, a mAb conjugate targeting CD20, was shown to greatly extend survival times in early- and late-stage lymphoma models compared to the untreated and non-specific controls (Durand-Panteix et al. 2021). CD37 targeting mAb construct, [^212^Pb]Pb-NNV003, demonstrated similar efficacy in models of chronic lymphocytic leukaemia and non-Hodgkin’s lymphoma, and CD38 was likewise an effective target for [^212^Pb]Pb-daratumumab in improving survival in the treatment of multiple Myeloma (Maaland et al. 2020; Quelven et al. 2020). Only transient haematological activity was observed in the latter two studies, though it was noted that NNV003 and daratumumab do not bind to the murine CD38 or CD37 isotypes, and thus may have a different toxicity profile in humans.

In the systemic treatment of solid tumours, varying results have been reported. A ^212^Pb conjugated melanin targeting mAb was as efficacious as an untargeted mAb conjugate in the treatment of a murine melanoma model (Jiao et al. 2023). This was speculated to be the result of the ‘antigen-barrier’ effect whereby the presence of an antigen at the tumour surface, in this case melanin, sequesters the mAb, preventing it from penetrating the tumour. Due to the short range of α particles, this effectively prevents irradiation of the internal tumour volume. In contrast, ^212^Pb-labeled antibodies targeting CD46 and Chondroitin Sulphate Proteoglycan 4 (CSP4) substantially reduced tumour volumes in prostate and breast cancer mouse models respectively (Kasten et al. 2018b; Li et al. 2023a). Notably, the biodistribution of the CSP4 targeting construct was profoundly affected by the initial size of the tumour. Given the promising results treating smaller, vascularised tumours (VCAM-1 & lymphoid targeting therapies) compared to the relative difficulty with melanin targeted melanoma treatments, i.v. administered mAb targeted therapy appears to be profoundly influenced by the tumour environment.

#### Antibodies fragments and pretargeting

The key advantage of antibodies is their ability to specifically bind to almost any biological target with a high affinity (Ministro et al. 2020). As noted in the previous section however, their pharmacokinetic properties have been a hinderance to their adoption, and currently no antibody-targeted ^212^Pb treatments are being investigated clinically. The ideal targeting vector would have the exquisite binding properties of an antibody, but the rapid pharmacokinetic properties of a small molecule or peptide. To this end, two strategies have been implemented to improve antibody targeted ^212^Pb therapy.

The first is to use antibody fragments. Since only the paratope is strictly necessary for target binding, fragments containing this region can be used instead of the whole antibody (Tsai and Wu 2018). Smaller antibody fragments generally exhibit faster pharmacokinetics and more ready penetrate tissue, albeit at the cost of decreased affinity and stability (Wei et al. 2024). The decrease in affinity may be advantageous in some cases, as it could help overcome the “antigen-barrier” effect previously mentioned (Jiao et al. 2023). The only such reported study with ^212^Pb utilised the F(ab′)_2_ fragment derived from panitumumab, an EGFR targeting antibody (Milenic et al. 2018). In contrast to the intact immunoglobulin, the ^203^Pb-F(ab**'**)_2_ conjugate had a similar biodistribution when administered intravenously or intraperitoneally. Administration of the ^212^Pb-F(ab′)_2_ conjugate via either route was effective at increasing survival times in a peritoneal colorectal tumour model by over fivefold. A cooperative survival benefit was also observed combining i.v. administration of the ^212^Pb-F(ab′)_2_ conjugate with i.p. administration of the chemotherapeutic agents paclitaxel and gemcitabine. Evidently, this approach is a promising alternative to conventional mAb targeted therapy, and more research is needed to explore the range of antibody fragments available for use with ^212^Pb.

Antibody based therapy may also be improved by adopting a pre-targeting approach, whereby the antibody and radionuclide are administered separately, then combine in vivo (Altai et al. 2017). After administration of the antibody, sufficient time is allowed for tumour accumulation and clearance from circulation. The radionuclide is then administered, which is conjugated to a small targeting vector that specifically binds to the antibody. The radionuclide thus accumulates in the tumour, with excess being rapidly cleared. In this way, the targeting properties of antibodies are effectively combined with the pharmacokinetics of small molecules. With ^212^Pb, this approach was first investigated with a streptavidin-mAb targeting vector, and a ^212^Pb-labelled biotin radio-conjugate (Su et al. 2005). In healthy mice, the ^212^Pb-biotin complex was rapidly cleared, while in tumour bearing mice ^212^Pb readily accumulated in the tumours with little uptake observed in any other organ. However, the high immunogenicity of the biotin-streptavidin system currently poses a major limitation on its clinical use (Jallinoja and Houghton 2021). An alternative strategy was employed in a recent study, where a *trans-*cyclooctene modified antibody was administered alongside a ^212^Pb-labeled 1,2,4,5-tetrazine (Bauer et al. 2024). These components selectively undergo an inverse electron demand Diels–Alder reaction in vivo, covalently binding them together without the immunogenicity of the biotin-streptavidin system. Rapid tumour uptake and clearance were observed following administration of the ^212^Pb conjugate, and a dose dependent reduction in tumour volume and increase in survival was demonstrated in a pancreatic adenocarcinoma model.

#### Dual targeting with ^224^Ra

^224^Ra has similar radiochemical properties to the clinically approved ^223^Ra, and therefore also functions as an α-emitting bone-seeking agent (Juzeniene et al. 2018; Schumann et al. 2018). A ^212^Pb radiopharmaceutical can thus be administered alongside its ^224^Ra parent, allowing dual targeting of both cancerous cells, and skeletal metastases (Juzeniene et al. 2022). One such approach employed a solution of ^224^Ra administered with the phosphonate chelator EDTMP (Fig. 3b), which facilitated the localisation of the ^212^Pb progeny to the bone (Juzeniene et al. 2018). In a murine osteolytic breast cancer metastasis model, the ^224^Ra EDTMP solution exhibited a dose dependent reduction in the number of metastatic lesions, and extended mean survival times. Another application of this strategy is to label a radiopharmaceutical with ^212^Pb using a solution of ^224^Ra at equilibrium with its daughters, which is then administered without prior removal of ^224^Ra. The feasibility of this approach was demonstrated with the PSMA targeting ligand NG001 (Fig. 6), which could be readily radiolabelled with ^212^Pb in the ^224^Ra solution, and subsequently administered (Stenberg et al. 2020a). As expected, [^212^Pb]Pb-NG001 and ^224^Ra exhibited tumour and skeletal uptake respectively in a murine prostate cancer model, though no efficacy studies were performed. In a similar study, a ^224^Ra solution was used to prepare [^212^Pb]Pb-TCMC-TP-3, a murine IgG antibody which binds to cell surface antigen overexpressed by osteosarcomas (Tornes et al. 2022). In a multicellular spheroid model of metastatic osteosarcoma, the ^224^Ra and [^212^Pb]Pb-TCMC-TP-3 solution effectively inhibited the growth of the model tumour, warranting further investigation. While this dual targeting strategy has shown promise, ^212^Pb and ^212^Bi produced from ^224^Ra in vivo is likely to redistribute, and contribute to organ toxicity. Future studies are thus required to evaluate the efficacy, and toxicity of this approach.

### Imaging of ^212^Pb

Evaluating the dosimetry and dose–response of a radiopharmaceutical requires accurate biodistribution information. This is typically provided by nuclear imaging techniques such as SPECT or position emission tomography (PET) (Crișan et al. 2022). In SPECT imaging, γ-photons are detected by γ-cameras, which take a series of planar images that are used to generate a 3-dimensional image. Importantly, SPECT can only image photons within a specific energy range, generally 100–400 keV. In contrast, PET imaging detects pairs of 511 keV photons, which are emitted in opposite directions from electron–positron annihilation. The source of these annihilation events can then be localised and reconstructed to generate a 3-dimensional image. PET produces higher quality and more easily quantified images than SPECT, though it requires the use of a positron emitting nuclide (Crișan et al. 2022; O’Donoghue et al. 2022).

#### Surrogate imaging

Many therapeutic nuclides cannot be directly imaged by these techniques, as they do not emit positrons, nor photons suitable for SPECT. Imaging α-emitting nuclides is particularly challenging, as they are administered at relatively low levels of activity. Thus, it is common to use an easily imaged radionuclide as a surrogate to predict the biodistribution of radiopharmaceutical. PET emitters such as ^68^ Ga and ^64^Cu are typically employed for this purpose, both of which have been used with ^212^Pb (Bauer et al. 2024; Delpassand et al. 2022). However, different elements may have different pharmacokinetics even when conjugated to the same targeting vector, thus care must be taken to validate the distribution of a surrogate nuclide prior to dosimetry calculations (Brom et al. 2010; Nicolas et al. 2017).

A more common approach is to use ^203^Pb as an elementally equivalent ^212^Pb surrogate (Graves et al. 2024; McNeil et al. 2023). ^203^Pb, which can be readily produced via cyclotron irradiation of thallium targets, decays via electron capture to emit a 279 keV photon that can be easily imaged via SPECT (McNeil and Ramogida 2024; Nelson et al. 2023). Furthermore, its 52-h half-life allows the distribution of ^212^Pb to be predicted at extended time points. ^203/212^Pb have thus been dubbed a theranostic pair, and ^203^Pb has accordingly been widely used as a ^212^Pb surrogate in chelator design, target verification, biodistribution studies, and dosimetry calculations (dos Santos et al. 2019; Máthé et al. 2016; McDonagh et al. 2021; McNeil et al. 2021; Nelson et al. 2023). The main limitation of ^203^Pb imaging is that it does not account for differences in distribution between ^212^Pb and its progeny. Notwithstanding the percentage of ^212^Bi that dissociates, even where the complex remains intact the resulting ^212^Bi-labeled ligand may have a different pharmacokinetic profile than its ^212^Pb-labeled parent. This introduces considerable uncertainties when applying this information to inform the dosimetry of ^212^Pb. Alterative imaging technologies are thus being investigated to overcome this limitation.

#### Direct imaging

As most of the energy emitted by the ^212^Pb decay chain is produced by its α-emitting progeny, the distribution of both ^212^Pb and its daughters must be known to ensure accurate dosimetry calculations. Furthermore, since a percentage ^212^Bi dissociates from chelation following ^212^Pb decay, the use of radiopharmaceuticals labelled with alternative surrogates for the ^212^Pb daughters would not necessarily give an accurate picture of ^212^Pb dosimetry, since such a strategy does not account for ‘free’ ^212^Bi. Thus, an ideal modality for imaging ^212^Pb would be capable of imaging ^212^Pb and its daughters directly. Of the ^212^Pb decay chain nuclides, only ^212^Pb itself emits photons of sufficient energy and abundance for SPECT (Kvassheim et al. 2022). Preclinical studies have confirmed the utility of ^212^Pb SPECT, and demonstrated that quantitative SPECT/CT imaging may be feasible (Kvassheim et al. 2022; Kvassheim et al. 2023). Clinical ^212^Pb SPECT/CT images have also since been published (Griffiths et al. 2024; Michler et al. 2024). However, ^212^Pb SPECT again does not account for the distribution of progeny nuclides. Furthermore, the resolution and sensitivity of ^212^Pb-SPECT imaging remains poor, due to the low activities administered clinically, and scattering produced by the 2.6 MeV γ-emission of its ^208^Tl progeny (Kvassheim et al. 2022). This makes imaging of smaller and widely distributed tumours challenging, and thus ^212^Pb SPECT remains primarily a tool for qualitative distribution information.

Alternative nuclear imaging techniques exist which may be capable of imaging the ^212^Pb daughters. Both ^212^Bi and ^208^Tl generate imageable Cherenkov luminescence (CL) from their β^−^ particle emissions (Ackerman and Graves 2012; Wood and Ackerman 2016). CL imaging has been used preclinically to image ^212^Pb in vivo, and recent advancements in CL tomography may allow accurate three-dimensional distribution information to be obtained (Bauer et al. 2024; Wang et al. 2022b). However, CL imaging is currently limited by its lack of sensitivity. CL is emitted in the near-infrared to visible region of the electromagnetic spectrum, and thus is readily attenuated by tissue. Despite improvements in camera technology, imaging anything beyond superficial tumours remains a significant challenge, and hence CL imaging has been restricted primarily to preclinical studies (Mc Larney et al. 2021). Finally, Compton imaging is an emerging technique, theoretically capable of imaging the high energy γ-emissions of ^212^Bi and ^208^Tl. Originally developed for astronomy, Compton imaging can detect high energy (> 0.5 MeV) photons with a high degree of sensitivity and resolution (Kim and Lee 2024; Tashima and Yamaya 2022). Compton cameras are capable of imaging multiple radionuclides simultaneously, potentially allowing the distribution of ^212^Pb and its daughters to be quantitated and differentiated in real time (Sakai et al. 2018). However, biomedical Compton imaging remains in a developmental stage, and few studies have been conducted in vivo (Parajuli et al. 2022). With continued development though, Compton imaging may become a useful tool for imaging therapeutic radionuclides, including ^212^Pb.

## Conclusions

As a parent of α-emitting ^212^Bi, ^212^Pb continues to garner attention for use in TRT. Its favourable chemistry, half-life, and absence of long-lived daughters make it extremely attractive clinically, as does the presence of a readily available, elementally equivalent diagnostic radionuclide in ^203^Pb. Clinical research on ^212^Pb was historically hindered due to its limited supply, and thus it has received less attention than other α-emitters such as ^213^Bi, ^223^Ra, and ^225^Ac. However, ^212^Pb is now more available than ever, thanks to growing commercial interest, and the development of new generator technologies. This has sparked a surge of research in the field, exemplified by the numerous ^212^Pb based therapies now making their way to the clinic. This therapeutic application of ^212^Pb has largely been possible thanks to the decades-long effort to develop suitable chelating agents. TCMC particularly has enabled the rapid and stable radiolabelling of a range of targeting vectors, and has remained the standard chelator for ^212^Pb since its development over 20 years ago. Despite this, alternative chelators continue to be produced to improve radiolabelling and pharmacokinetic properties. With the clinical adoption of PSC, the utility of these alternate chelators is beginning to be realised, and one can expect to see more of them used in future studies. While the use of nanoconstructs represent an alternative to chelation, they remain underutilised and poorly studied with ^212^Pb TRT. Further research is needed to determine if these nanoconstructs are clinically viable.

^212^Pb TRT has demonstrated efficacy against a range of cancers and is well tolerated by patients. ^212^Pb has also consistently exhibited a cooperative effect when combined with other therapies, thus demonstrating the potential of its use alongside conventional treatment regimes. Peptidic and small molecule targeting vectors have been especially efficacious, with all ongoing clinical studies utilising such a targeting strategy. On the other hand, antibody-based ^212^Pb TRT remains a more nascent field despite being first to the clinic. While ^212^Pb labelled mAbs have shown the most promise in the treatment of peritoneal cancers, they have not yet demonstrated efficacy clinically. Systemic mAb therapy has been less promising, as their long circulating half-life produces high dosimetric loads on vital organs. These limitations may be overcome by using antibody fragments or a pre-targeting approach instead. The range of targeting vectors investigated for ^212^Pb TRT remains somewhat limited, with most developed first for use with other radionuclides. Future research should thus include the development of novel ligands that are optimised for use with ^212^Pb, by targeting cancers and disease presentations that are well suited to its unique half-life and decay characteristics. In any case, the continued development of imaging technologies and methodology will enable more accurate dosimetry, and thus further improve therapeutic outcomes.

Two main limitations remain for the use of ^212^Pb, namely the dosimetric and personnel hazard of the 2.6 MeV ^208^Tl γ-emission, and the dissociation of ^212^Bi following ^212^Pb decay. In either case however, strategies exist to mitigate these issues. Radiation exposure to personnel can be minimised with dedicated production facilities which offer adequate shielding, and automated processes to minimise handling of radioactive material. Well-designed preclinical and clinical studies can assess the dosimetric load of this emission on the patient, and optimal doses can be established accounting for it. Similarly, renal toxicity of released ^212^Bi has been mitigated for the most part by locoregional administration and/or targeting internalisng receptors, thereby preventing ^212^Bi redistribution. Beyond this, increasing clearance though adjuvants, or the use of alterative ^212^Pb carriers may also be effective. In any case, ^212^Pb undoubtably possesses therapeutic potential, which will only be fully realised with further research.

## Data Availability

Not applicable.

## Abbreviations

B7-H3: B7 homolog 3 protein
CD146: Cluster of differentiation 146
CL: Cherenkov luminescence
CSP4: Chondroitin sulphate proteoglycan 4
CXCR4: Chemokine receptor type 4
DMPS: 2,3-Dimercapto-1-propanesulfonic acid
DOTA: 1,4,7,10-Tetraazacyclododecane-1,4,7,10-tetraacetic acid
DTPA: Diethylenetriaminepentaacetic acid
EGFR: Epidermal growth factor receptor
FAP: Fibroblast activation protein
GPCR: G-protein coupled receptor
GRPr: Gastrin releasing peptide receptor
HER2: Human epidermal growth factor receptor 2
i.p.: Intra-peritoneal
i.v.: Intravenous
mAb: Monoclonal antibody
MC1R: Melanocortin receptor 1
PET: Position emission tomography
PSC: Pb specific chelator
PSMA: Prostate specific membrane antigen
PTK7: Protein tyrosine kinase 7
SPECT: Single-photon emission computed tomography
SSTR2: Somatostatin receptor subtype 2
TCMC: 1,4,7,10-Tetraazacyclododecane-1,4,7,10-tetraacetic amide
TRT: Targeted Radionuclide Therapy
VCAM-1: Vascular cell adhesion molecule 1
α-MSH: α-Melanocyte stimulating hormone
